# Sex differences in the inflammatory response of primary astrocytes to lipopolysaccharide

**DOI:** 10.1186/2042-6410-2-7

**Published:** 2011-07-11

**Authors:** María Santos-Galindo, Estefanía Acaz-Fonseca, María J Bellini, Luis M Garcia-Segura

**Affiliations:** 1 Instituto Cajal, CSIC, Avenida Doctor Arce 37, E-28002 Madrid, Spain

**Keywords:** IFN-inducible protein 10, IL1β, IL6, steroidogenic acute regulatory protein, testosterone, Toll-like receptor 4, translocator protein 18 kDa, TNFα

## Abstract

**Background:**

Numerous neurological and psychiatric disorders show sex differences in incidence, age of onset, symptomatology or outcome. Astrocytes, one of the glial cell types of the brain, show sex differences in number, differentiation and function. Since astrocytes are involved in the response of neural tissue to injury and inflammation, these cells may participate in the generation of sex differences in the response of the brain to pathological insults. To explore this hypothesis, we have examined whether male and female astrocytes show a different response to an inflammatory challenge and whether perinatal testosterone influences this response.

**Methods:**

Cortical astrocyte cultures were prepared from postnatal day 1 (one day after birth) male or female CD1 mice pups. In addition, cortical astrocyte cultures were also prepared from female pups that were injected at birth with 100 μg of testosterone propionate or vehicle. Cultures were treated for 5 hours with medium containing lipopolysaccharide (LPS) or with control medium. The mRNA levels of IL6, interferon-inducible protein 10 (IP10), TNFα, IL1β, Toll-like receptor 4 (TLR4), steroidogenic acute regulatory protein and translocator protein were assessed by quantitative real-time polymerase chain reaction. Statistical significance was assessed by unpaired *t*-test or by one-way analysis of variance followed by the Tukey *post hoc *test.

**Results:**

The mRNA levels of IL6, TNFα and IL1β after LPS treatment were significantly higher in astrocytes derived from male or androgenized females compared to astrocytes derived from control or vehicle-injected females. In contrast, IP10 mRNA levels after LPS treatment were higher in astrocytes derived from control or vehicle-injected females than in those obtained from males or androgenized females. The different response of male and female astrocytes to LPS was due neither to differences in the basal expression of the inflammatory molecules nor to differences in the expression of the LPS receptor TLR4. In contrast, the different inflammatory response was associated with increased mRNA levels of translocator protein, a key steroidogenic regulator, in female astrocytes that were treated with LPS.

**Conclusions:**

Male and female cortical astrocytes respond differentially to an inflammatory challenge and this may be predetermined by perinatal testosterone exposure.

## Background

Astrocytes, one of the glial cell types of the central nervous system (CNS), are involved in a variety of functions under physiological conditions, including the control of brain blood flow and neuronal metabolism [1,2]. In addition, astrocytes regulate extracellular potassium levels and neuronal excitability and, by means of astrocyte-astrocyte and astrocyte-neuron communication, participate in the regulation of synaptic transmission, synaptic plasticity and information processing in the CNS [3,4]. Astrocytes also play an important role under pathological conditions. Together with microglia, these cells participate in the local inflammatory response of the CNS, releasing a variety of inflammatory mediators, including cytokines, such as IL6, TNFα and IL1β, and chemokines, such as IFN-inducible protein 10 (IP10) [5-7]. Astrocytes also express Toll-like receptor 4 (TLR4), which mediates the inflammatory actions of lipopolysaccharide (LPS) in these cells [8-12].

Sex differences in the number, differentiation and function of astrocytes [13-24] are associated in several CNS regions with sex differences in the structure and function of neuronal circuits [17,25,26] and are thought to participate in the generation of sex differences in neuroendocrine regulation, behavior and cognition. In addition, sex differences in astrocytes are relevant under pathological conditions. Numerous neurological and psychiatric disorders, including Parkinson's disease, Alzheimer's disease, Huntington's disease, multiple sclerosis, traumatic brain injury, stroke, autism, schizophrenia, depression, anxiety disorders, eating disorders and peripheral neuropathy, show sex differences in incidence, age of onset, symptomatology and outcome [27-31]. It is plausible that sex differences in astrocytes might be involved in the generation of sex differences in the manifestation of brain pathological alterations. In this regard, previous studies have shown that cortical astrocytes from male mice are less resistant than those from female mice to oxygen-glucose deprivation [32]. This sex difference may be due to different activity of the enzyme aromatase, which converts testosterone into estradiol; aromatase activity is higher in cortical female astrocytes than in cortical male astrocytes [32]. Since aromatase activity is neuroprotective [33,34], the sexually dimorphic activity of the enzyme in astrocytes may contribute to protect not only these cells but also other CNS cell types, including neurons, from damage.

Furthermore, astrocytes express other enzymes involved in steroid synthesis and metabolism [35] and release a variety of neuroprotective steroids, such as pregnenolone, progesterone and its reduced metabolites, dihydroprogesterone and tetrahydroprogesterone [36-42], which may influence the outcome of neurodegenerative diseases. In addition, astrocytes express steroidogenic acute regulatory protein (StAR) and translocator protein of 18 kDa (TSPO, previously known as peripheral benzodiazepine receptor) [43-45]. These proteins are involved in the first step of steroidogenesis: the conversion of cholesterol into pregnenolone by the enzyme P450scc. This step is highly regulated by the activity of several proteins, such StAR and TSPO, which facilitate the transport of cholesterol through the hydrophilic space located between the outer and inner mitochondrial membranes, where P450scc is located [45,46].

While the responses of astrocytes to pathological conditions are well documented, the possible existence of sex differences in these responses has received less attention. Therefore, in this study, we have assessed whether cortical astrocytes derived from male and female mice have a different reaction to an inflammatory challenge induced by LPS and whether this is associated with differences in the expression of two main steroidogenic regulators: StAR and TSPO. We have assessed the expression of IL6, TNFα, IL1β and IP10. These molecules were selected because their expression is altered in the CNS under different pathological conditions, including Alzheimer's disease, Parkinson's disease, multiple sclerosis and traumatic brain injury [7]. We have also explored whether the perinatal peak of androgens, which participate in the sex differentiation of the CNS in rodents [47], may influence the subsequent inflammatory response of astrocytes to LPS. Our results indicate that cortical primary astrocytes derived from male and female mice pups show different expression of inflammatory markers in response to LPS. This difference is associated with increased expression of TSPO in female astrocytes exposed to LPS. In addition, our findings suggest that perinatal testosterone might be involved in the sexually dimorphic inflammatory response of astrocytes.

## Results

### Astrocytes from males and females expressed different mRNA levels of IL6, IP10, TNFα and IL1β after LPS exposure

**Figure 1:**
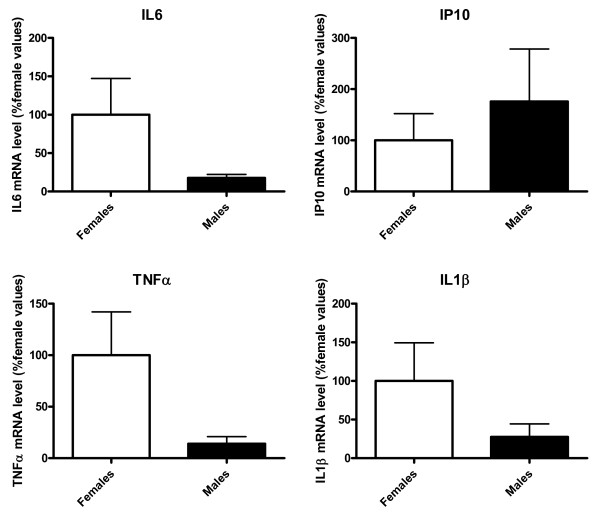
**Male and female astrocytes express similar mRNA levels of interleukin 6, IFN-inducible protein 10, TNFα and interleukin 1β**. mRNA levels of interleukin 6 (IL6), IFN-inducible protein 10 (IP10), TNFα and interleukin 1β (IL1β) in cortical astrocyte cultures from male and female mouse pups under basal conditions are shown. Data are means ± standard errors of the mean (SEM) and are expressed as percentages of female values. No statistically significant differences were detected.

The mRNA levels of IL6, IP10, TNFα and IL1β were first assessed in astrocytes from cultures not treated with LPS. The results indicate that, under basal conditions, cultures obtained from postnatal day 1 (PND1) male and female pups expressed similar mRNA levels of these cytokines (Figure 1).

**Figure 2:**
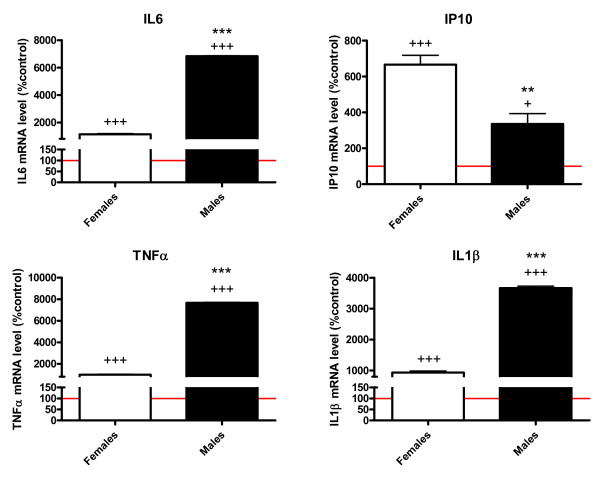
**Male and female astrocytes show a different response to LPS**. mRNA levels of IL6, IP10, TNFα and IL1β in lipopolysaccharide (LPS)-treated cortical astrocyte cultures from male and female mouse pups are shown. Data are means ± SEM and are expressed as percentages of controls (red line indicates astrocytes cultured with control medium without LPS). **P < 0.01 and ***P < 0.001, indicating significant differences compared with female values. +*P *< 0.05 and +++*P *< 0.001, indicating significant differences compared with cultures not treated with LPS.

LPS treatment increased the mRNA levels of IL6, IP10, TNFα and IL1β in the cultures from both male and female mice. However, the increase in mRNA levels of IL6, TNFα and IL1β was significantly higher in the cultures obtained from male pups. In contrast, the increase in IP10 mRNA levels was higher in the cultures obtained from females (Figure 2).

### Astrocytes from control and androgenized females expressed different mRNA levels of IL6, IP10, TNFα and IL1β after LPS exposure

**Figure 3:**
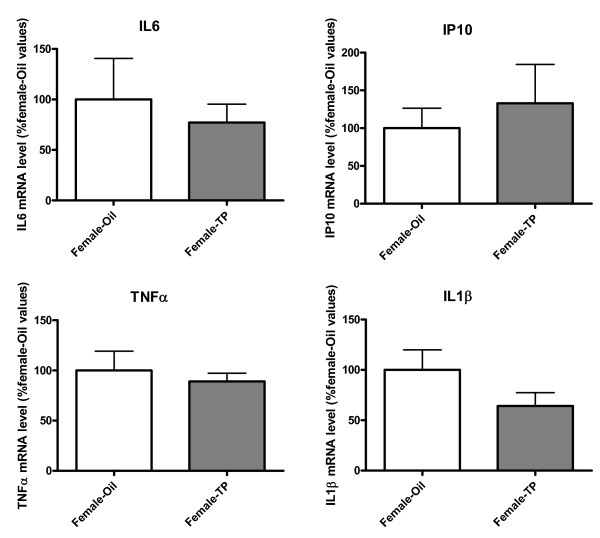
**Astrocytes from control and androgenized females express similar mRNA levels of IL6, IP10, TNFα and IL1β**. mRNA levels of IL6, IP10, TNFα and IL1β in cortical astrocyte cultures under basal conditions are shown. Cultures were prepared from postnatal day 1 (PND1) female mouse pups that were injected on PND0 with vehicle (Female-Oil) or testosterone propionate (Female-TP). Data are means ± SEM and are expressed as percentages of female oil values. No statistically significant differences were detected.

To explore the basis of the sex differences detected in the previous experiment, we obtained astrocyte cultures from females treated 24 hours earlier, on PND0, with testosterone propionate and from control females injected on PND0 with vehicle. No significant differences in the levels of IL6, IP10, TNFα and IL1β were detected under basal conditions (Figure 3).

**Figure 4:**
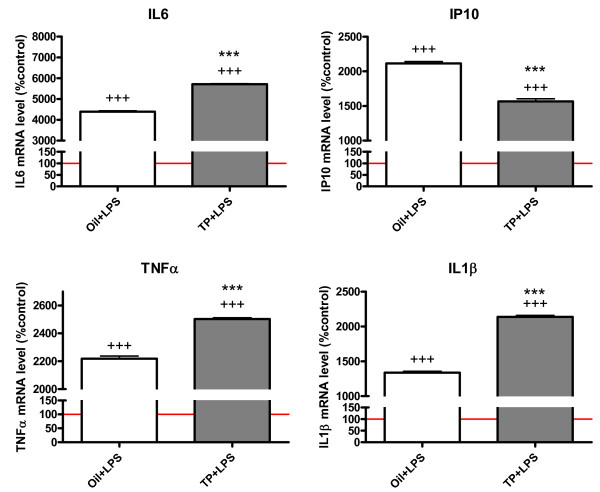
**Astrocytes from control and androgenized females show a different response to LPS**. mRNA levels of IL6, IP10, TNFα and IL1β in LPS-treated cortical astrocyte cultures from female mouse pups injected with oil (Oil+LPS) and from female mouse pups treated with testosterone propionate (TP+LPS). Data are means ± SEM and are expressed as percentages of control values (red line indicates astrocytes cultured with control medium without LPS). ***P < 0.001, indicating significant differences compared with values of female mouse pups treated with oil. +++*P *< 0.001, indicating significant differences compared with cultures not treated with LPS.

LPS treatment increased the mRNA levels of IL6, IP10, TNFα and IL1β in all cultures. However, the increase in mRNA levels of IL6, TNFα and IL1β was higher in the cultures obtained from androgenized females, whereas the increase in IP10 mRNA levels was higher in the cultures obtained from control females (Figure 4).

### Astrocytes from males and females expressed similar mRNA levels of TLR4 under basal conditions and after LPS exposure

**Figure 5:**
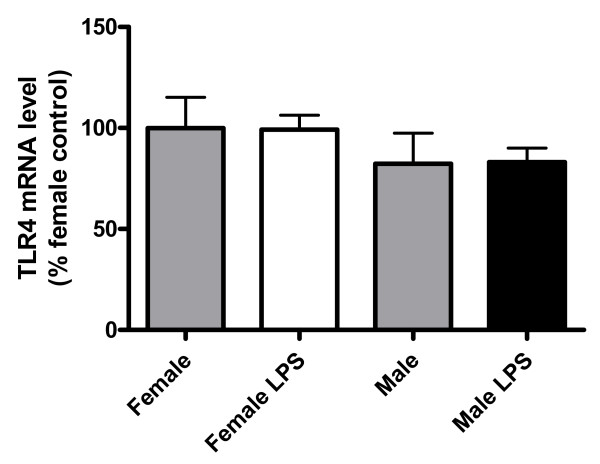
**Astrocytes from males and females express similar levels of TLR4**. mRNA levels of Toll-like receptor 4 (TLR4) in control and LPS-treated cortical astrocyte cultures from female and male mouse pups. Data are means ± SEM and are expressed as percentages of control female astrocytes.

To determine whether the sexually dimorphic response to LPS was due to different expression levels of the LPS receptor TLR4 in male and female astrocytes, we measured the mRNA levels of TLR4 in control and LPS-treated astrocyte cultures. No significant differences were detected in the basal mRNA levels of TLR4 between the cultures derived from male and female pups. In addition, TLR4 mRNA levels were not significantly affected by treatment with LPS (Figure 5).

### Astrocytes from males and females showed different regulation of TSPO mRNA levels after LPS exposure

**Figure 6:**
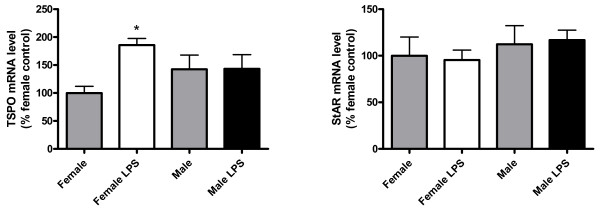
**Male and female astrocytes show a different regulation of TSPO levels after LPS exposure**. mRNA levels of translocator protein 18 kDa (TSPO) and steroidogenic acute regulatory protein (StAR) in control and LPS-treated cortical astrocyte cultures from female and male mouse pups. Data are means ± SEM and are expressed as percentages of control female astrocytes. *P < 0.05, indicating significant difference compared with control female values.

To determine whether the sex differences observed in the response of IL6, IP10, TNFα and IL1β mRNA levels to LPS were associated with sex differences in the expression of steroidogenic proteins, we assessed the mRNA levels of two proteins involved in cholesterol transport to the mitochondria: TSPO and StAR. No significant differences were detected in the mRNA levels of TSPO and StAR between cultures from males and females under basal conditions. However, LPS increased the mRNA levels of TSPO in the cultures from females, but not in the cultures from males. LPS did not affect StAR mRNA levels (Figure 6).

## Discussion

In this study, we have assessed the expression of IL6, IP10, TNFα and IL1β by cultured astrocytes derived from the cerebral cortices of neonatal male and female mice. The mRNA levels of these inflammatory markers increased in both male and female astrocytes after incubation with LPS. These findings are in agreement with previous studies showing that LPS induces an inflammatory response in cultured astrocytes [5,12,48]. However, our present results indicate that male and female astrocytes differ in the characteristics of their inflammatory response. In response to LPS, male astrocytes showed enhanced expression of IL6, TNFα and IL1β, while female astrocytes showed enhanced expression of IP10. In contrast, under basal unstimulated conditions, astrocytes from males and females showed similar mRNA levels of IL6, IP10, TNFα and IL1β. This suggests that it is the response of astrocytes to LPS that is sexually dimorphic, not the mechanisms that regulate the basal expression of IL6, TNFα, IL1β and IP10.

The inflammatory response to LPS is mediated by TLR4, a member of the IL1 receptor/TLR superfamily that is expressed by astrocytes [8-12]. Our findings indicate that TLR4 mRNA levels are not significantly different between male and female astrocytes both under resting conditions and after LPS treatment, suggesting that the sex difference in the inflammatory response of astrocytes is not mediated by different expression of LPS receptors. However, we cannot exclude differences in TLR4 signaling between male and female astrocytes as a mediating factor}.

Sex differences in the manifestation of several neurodegenerative diseases are associated with differences in local steroid levels in the CNS [49,50]. Astrocytes express proteins such as TSPO that are involved in the transport of cholesterol from the outer to the inner mitochondrial membrane, where P450scc, the first enzyme of the steroidogenic pathway, is located [35,51]. Therefore, astrocytes can convert cholesterol in pregnenolone by the enzyme P450scc. In addition, rodent astrocytes express all the steroidogenic enzymes necessary to metabolize pregnenolone in other steroids, such as progesterone and estradiol [35]. Since some of the steroids derived from pregnenolone have anti-inflammatory properties and reduce astrogliosis [52-55], and since TSPO expression is increased after different forms of neural injury [56-58], we examined whether LPS could induce changes in TSPO expression in astrocytes. Previous studies have shown that TSPO is involved in the control of reactive gliosis [59,60] and regulates several mitochondrial functions, including the control of apoptosis [61,62], which may also affect the inflammatory response. Our findings indicate that LPS increases the levels of TSPO in cultured astrocytes derived from female mice, but not in astrocyte cultures derived from male mice. However, the mRNA levels of TSPO were not different between female astrocytes stimulated with LPS and male astrocytes under basal and LPS-stimulated conditions. Therefore, it is unclear whether the different responses of TSPO to LPS between male and female astrocytes are sufficient to explain the sexually dimorphic inflammatory responses of these cells.

We also analyzed the effect of LPS on the expression of StAR, another molecule involved in the transport of cholesterol to the mitochondria [44,45]. In the brain *in vivo*, StAR is expressed in specific populations of astrocytes and neurons, although most cells expressing the protein are neurons [45,63]. As for TSPO, previous studies have shown that StAR expression is increased in the CNS after injury [63,64]. However, in contrast to TSPO, LPS did not induce a change in the expression of StAR mRNA in astrocytes. In addition, we did not detect a difference in StAR expression between male and female astrocytes. However, we cannot exclude the possibility of differences in StAR activity, which is regulated by posttranslational modifications of the protein [45].

Sex differences in astrocytes can result from direct sex chromosome differences in these cells and/or from different exposure to sex steroids during development [65]. Previous *in vivo *studies have shown that perinatal actions of androgens are involved in the generation of sex differences in the expression of glial fibrillary acidic protein and in the differentiation and number of astrocytes in different brain regions [13,20,23,66]. Our findings indicate that astrocyte cultures derived from female pups that were androgenized on PD0 show a response to LPS similar to that of astrocyte cultures derived from male pups. Thus, LPS induced an increase in the mRNA levels of IL6, TNFα and IL1β in the cultures obtained from males and androgenized females compared to control females. In contrast, LPS induced an increase in IP10 mRNA levels in the cultures obtained from control females compared to the cultures obtained from males and androgenized females. Therefore, it seems that the action of testosterone on astrocytes on PD0 is able to produce modifications in these cells that are maintained even after their extraction from the brain and their regrowth *in vitro*.

Our findings showing sex differences in the inflammatory response of astrocytes are consistent with previous observations indicating that female astrocytes are more resistant than male astrocytes to oxygen-glucose deprivation and oxidant-induced cell death [32,67]. If translatable to *in vivo *conditions, the different *in vitro *inflammatory responses of male and female astrocytes detected in this study could contribute to sex differences in the manifestation of neurodegenerative diseases. For instance, different expression of IP10 between male and female astrocytes *in vivo *could result, in sex differences in the recruitment of T lymphocytes, natural killer cells and monocytes into the CNS [68,69]. Sex differences in the expression of IL1β by astrocytes *in vivo *could result in sex differences in neuronal injury [70] and in the remyelination of multiple sclerosis lesions [71]. Enhanced IL6 production by male astrocytes could result in sex differences in the outcome of acute and chronic pathological conditions, since astrocyte-driven production of IL6 *in vivo *increases chronic inflammatory damage [72] but protects the CNS against acute focal injury [73-75]. Finally, different production of TNFα by male and female astrocytes could result in sex differences in microglial reactivity [76], dysfunction of the blood-brain barrier [77] and the survival of oligodendrocyte precursors [78,79]. Therefore, further studies should explore the functional consequences of the different inflammatory responses of male and female astrocytes and whether this sexually dimorphic response affects astrocytes' survival, activation, proliferation and ability to protect neurons and oligodendrocytes under pathological conditions.

## Conclusions

In summary, our findings indicate that primary cortical astrocytes in mice respond differently to an inflammatory challenge, depending on whether they are obtained from males or females. In addition, primary astrocytes derived from perinatally androgenized females respond to inflammatory challenges in the same way that primary astrocytes derived from males do, suggesting that perinatal testosterone programs astrocytes for a different response to pathological conditions. These sex differences with regard to astrocytes may be relevant to the generation of sex differences in brain pathology.

## Methods

### Animals and *in vivo *treatments

CD1 mice dams from the Instituto Cajal animal colony were kept on a 12-hour light-dark schedule and received food and water *ad libitum *until birth. Male and female pups were distinguished by a larger genital papilla and longer anogenital distance in male pups than in female pups. Experimental procedures were approved by our Institutional Animal Use and Care Committee (Spanish National Research Council Animal Experimentation Committee). Special care was taken to minimize suffering and to reduce the number of animals used to the minimum required for statistical accuracy.

In a first set of experiments, PND1 male and female pups were used to obtain cortical astrocyte cultures. In a second set of experiments, female pups were injected at birth (PND0) with 50 μL of corn oil (vehicle) or 100 μg of testosterone propionate (diluted 2 mg/mL; Fluka Chemie, Buchs, Switzerland). Cortical astrocyte cultures were obtained from these females on PND1, 24 hours after the administration of testosterone propionate or vehicle. The subsequent processing was the same in both sets of experiments.

### Cortical astrocyte primary cultures and *in vitro *treatments

Astrocytes were cultured from PND1 male and CD1 female mice pups separately. The brain was extracted, the meninges were removed and the entire neocortex was isolated under a dissecting microscope. Next, the cortex was dissociated mechanically, washed twice in Hank's balanced salt solution (Invitrogen, Paisley, UK) and filtered through a 40-μm nylon cell strainer into phenol red-free DMEM-F12 (Invitrogen, Paisley, UK) containing 10% fetal bovine serum (FBS; Invitrogen) and penicillin-streptomycin (Invitrogen). After centrifugation at 1,000 rpm for 5 minutes, cells were resuspended and cultured in 75-cm^2 ^tissue culture flasks at 37°C and 5% CO_2_. The medium was changed after one day *in vitro *and subsequently two times per week until the cells reached confluence. Next, enriched astrocyte cultures were obtained after overnight shaking at 37°C and 280 rpm on a tabletop shaker (Thermo Forma, Marietta, OH, USA) to minimize oligodendrocyte and microglia contamination. Astrocytes were removed from the flasks by incubation with 0.5% trypsin (type II-S; Sigma-Aldrich, St Louis, MO, USA) and 0.04% ethylenediaminetetraacetic acid (Sigma-Aldrich). Trypsinization was stopped with DMEM-F12 medium with FBS, the cell suspension was centrifuged and the pellet was resuspended and plated onto poly-L-lysine-coated six-well plates in serum-free medium and used within 24 hours. One day after plating astrocytes were treated for 5 hours with medium containing LPS (500 ng/mL LPS, *Escherichia coli *026:B6; Sigma-Aldrich) or with control medium without LPS. The dose of LPS was based on that used in a previous study [5].

### Quantitative real-time polymerase chain reaction

After 5 hours of treatment with LPS, culture medium was removed and cells were lysed. Total RNA was extracted using the illustra RNAspin Mini RNA Isolation Kit (GE Healthcare, Buckinghamshire, UK) to assess the mRNA expression levels of IL6, IP10, TNFα, IL1β, TLR4, StAR and TSPO.

First-strand cDNA was prepared from 1 μg of RNA using M-MLV Reverse Transcriptase (Promega, Madison, WI, USA) according to the manufacturer's protocol. After reverse transcription, the cDNA was diluted 1:4 for the unknown gene and 1:100 for the endogenous control, and 5 μL of these cDNA solutions were amplified by real-time PCR in a 15 μL voulme reaction using SYBR Green PCR MasterMix (Applied Biosystems, Foster City, CA, USA) or TaqMan Universal PCR MasterMix (Applied Biosystems) using the ABI Prism 7500 Sequence Detection System (Applied Biosystems) with conventional Applied Biosystems cycling parameters (40 cycles of changing temperatures, first at 95°C for 15 seconds and then at 60°C for 1 minute). The primer sequences were designed using Primer Express software (Applied Biosystems) and are shown in Table 1.

**Table 1:** Primer sequences used for quantitative real-time polymerase chain reaction^a^

Gene	Forward Primer	Reverse Primer
IL6	5'-GAAACCGCTATGAAGTTCCTCTCTG-3'	5'-TGTTGGGAGTGGTATCCTCTGTGA-3'
IP10	5'-CAGTGAGAATGAGGGCCATAGG-3'	5'-CGGATTCAGACATCTCTGCTCAT-3'
TNFα	5'-GAAAAGCAAGCAGCCAACCA-3'	5'- CGGATCATGCTTTCTGTGCTC -3'
IL1β	5'- CGACAAAATACCTGTGGCCT -3'	5'- TTCTTTGGGTATTGCTTGGG -3'
StAR	5'-GTCCCTCCAAGACTAAACTCACTTG-3'	5'-GGTGGTTGGCGAACTCTATCTG-3'
TLR4	5'-GGCTCCTGGCTAGGACTCTGA-3'	5'-TCTGATCCATGCATTGGTAGGT-3'
TSPO	5'-TGCAGAAACCCTCTTGGCATC-3'	5'-TGAAACCTCCCAGCTCTTTCC-3'

^a^IL6, interleukin 6; IP10, IFN-inducible protein 10; TNFα, tumor necrosis factor α; IL1β, interleukin 1β; StAR, steroidogenic acute regulatory protein; TLR4, Toll-like receptor 4; TSPO, translocator protein of 18 kDa.

Glyceraldehyde 3-phosphate dehydrogenase (GAPDH) was selected as a control housekeeping gene. The GAPDH TaqMan probes and primers used were the Assays-on-Demand Gene Expression Products (Applied Biosystems). After amplification, a denaturing curve was performed to ensure the presence of unique amplification products.

To visualize and sequence the PCR products, each amplification product was electrophoresed on 2% (wt/vol) ethidium bromide-stained agarose gel. Next, bands were excised and cDNA was purified using the QIAquick PCR Purification Kit (Qiagen Iberia, Madrid, Spain). Samples were sequenced (Automatic Sequencing Center, CSIC, Madrid, Spain) with the corresponding forward or reverse primer. The obtained sequence was aligned with the expected sequence of each transcript obtained from GenBank. All reactions were performed in duplicate, and the quantities of target gene expression were normalized to the corresponding GAPDH expression in test samples and plotted.

### Statistical analysis

Statistical significance was assessed by performing an unpaired *t*-test for the one-to-one comparisons and one-way analysis of variance followed by the Tukey *post hoc *test for comparisons with more than two experimental groups. GraphPad Prism 5 software (GraphPad Software, San Diego, CA, USA) was used for the analysis. The significance level was set at *P *< 0.05. Data shown in the figures are the results of three to seven independent experiments and data are presented as the mean ± standard error of the mean.

## Abbreviations

CNS: central nervous system; DMEM: Dulbecco's modified Eagle's medium; IL: interleukin; IFN: interferon; IP10: IFN-inducible protein 10; LPS: lipopolysaccharide; PCR: polymerase chain reaction; StAR: steroidogenic acute regulatory protein; TLR4: Toll-like receptor 4; TNFα: tumor necrosis factor α; TSPO: translocator protein of 18 kDa.

## Competing interests

The authors declare that they have no competing interests.

## Authors' contributions

MSG and LMGS have designed the experiments and interpreted the data and have been involved in the writing of the manuscript. MSG, EAF and MJB have acquired the data and analyzed the experiments. All the authors have revised the manuscript and given final approval of the version to be published.
